# Supplementary material for: Transcriptome and Metabolome Analyses Reveal Mechanisms Underlying the Response of Quinoa Seedlings to Nitrogen Fertilizers
Source: Int J Mol Sci. 2023 Jul 18;24(14):11580. doi: 10.3390/ijms241411580 (PMC10380953; doi:10.3390/ijms241411580)
Supplement: Supplementary file 1 [file ijms-24-11580-s001.zip › Figure S1.pdf]

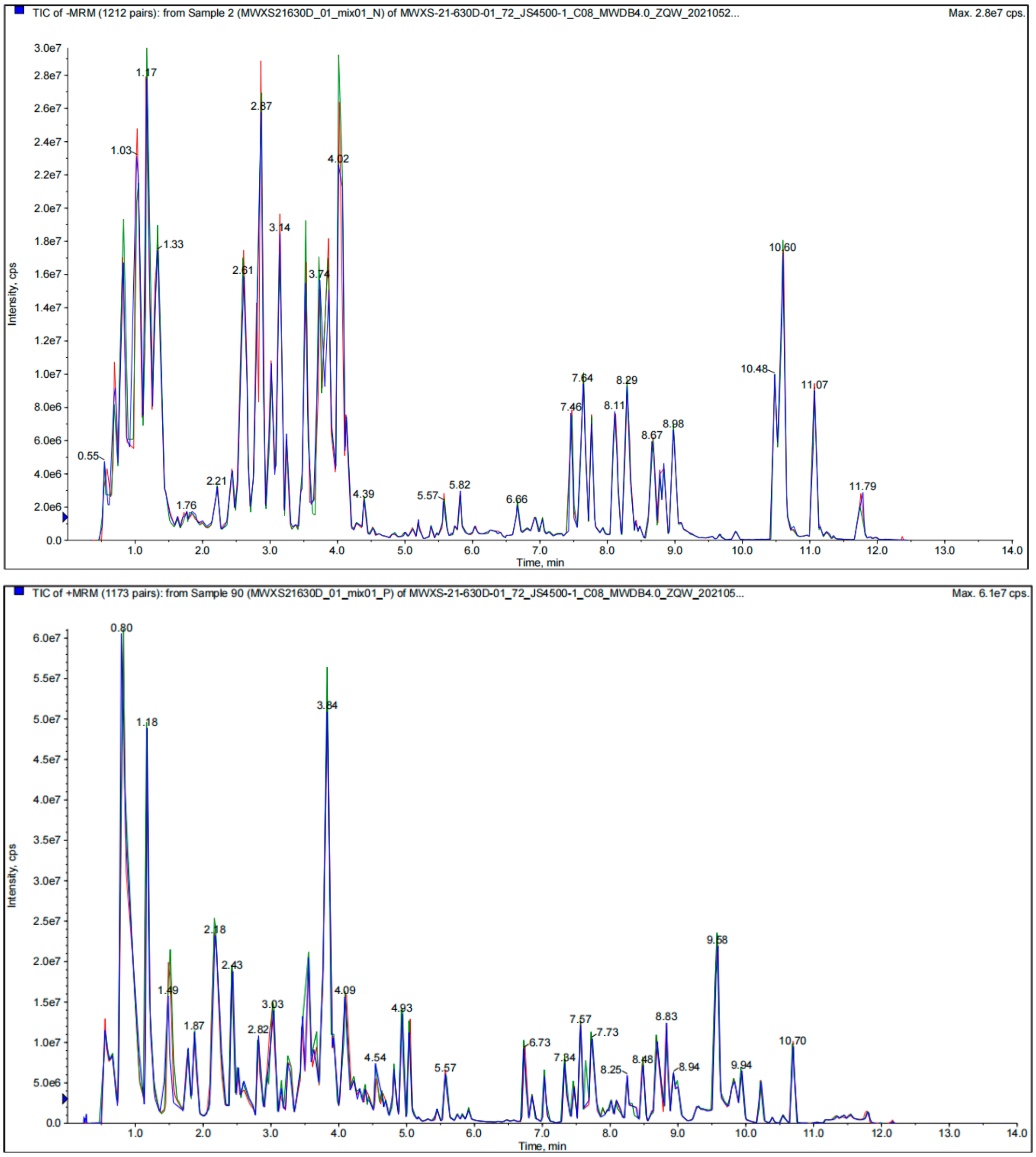

Figure S1 Total ion flow chromatogram in negative ion mode and positive ion mode. The multi-reaction monitoring (MRM) mode metabolite detection multipeak diagram shows the substances that can be detected in the sample. Each mass spectrum peak with different colors represents a detected metabolite. The characteristic ions of each substance are screened out through the triple four-stage rod, and the signal strength (CPS) of the characteristic ions is obtained in the detector. The sample of the machine mass spectrometry file is opened with the MultiQuant software to integrate and correct the chromatographic peaks. Each chromatographic peak represents the relative content of the corresponding substance.
