# Supplementary material for: Transcriptome and Metabolome Analyses Reveal Mechanisms Underlying the Response of Quinoa Seedlings to Nitrogen Fertilizers
Source: Int J Mol Sci. 2023 Jul 18;24(14):11580. doi: 10.3390/ijms241411580 (PMC10380953; doi:10.3390/ijms241411580)
Supplement: Supplementary file 1 [file ijms-24-11580-s001.zip › Figure S2.pdf]

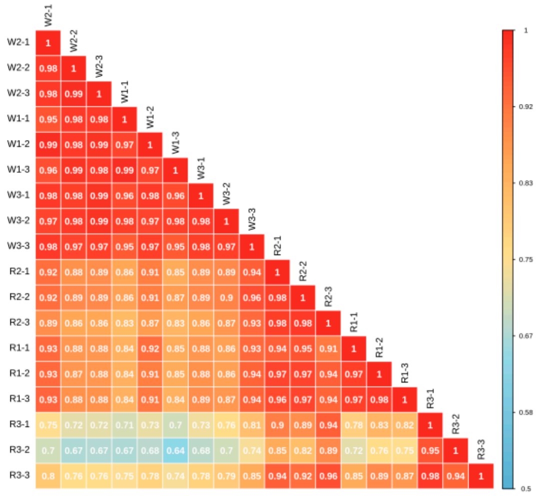

Figure S2 Correlation diagram between samples; the longitudinal and diagonal lines respectively represent the names of different samples. Different colors represent different Pearson correlation coefficients. The redder the color is, the stronger the correlation is. The bluer the color is, the worse the correlation is. At the same time, the correlation coefficients between the two samples are marked in the square.
