# Supplementary material for: Transcriptome and Metabolome Analyses Reveal Mechanisms Underlying the Response of Quinoa Seedlings to Nitrogen Fertilizers
Source: Int J Mol Sci. 2023 Jul 18;24(14):11580. doi: 10.3390/ijms241411580 (PMC10380953; doi:10.3390/ijms241411580)
Supplement: Supplementary file 1 [file ijms-24-11580-s001.zip › Figure S3.pdf]

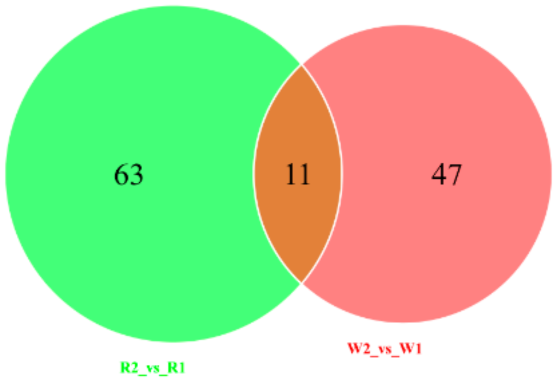

(a)

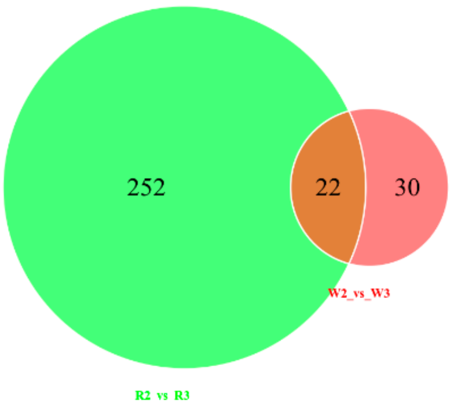

(b)

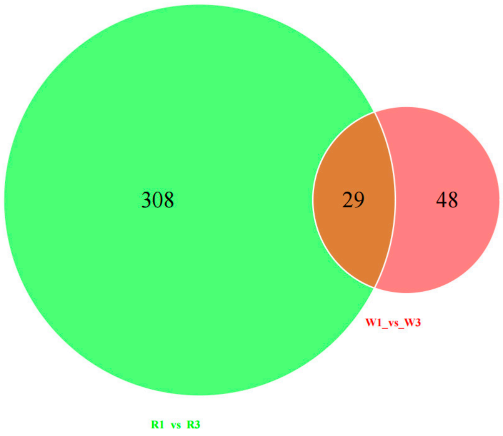

(c)

Figure S3 (a) Venn diagram of DEMs for CK-R vs. LN-R and CK-W vs. LN-W. **(b)** Venn diagram of DEMs for CK-R vs. HN-R and CK-W vs. HN-W. **(c)** Venn diagram of DEMs for LN-R vs. HN-R and LN-W vs. HN-W.
