# Supplementary material for: Transcriptome and Metabolome Analyses Reveal Mechanisms Underlying the Response of Quinoa Seedlings to Nitrogen Fertilizers
Source: Int J Mol Sci. 2023 Jul 18;24(14):11580. doi: 10.3390/ijms241411580 (PMC10380953; doi:10.3390/ijms241411580)
Supplement: Supplementary file 1 [file ijms-24-11580-s001.zip › Figure S4.pdf]

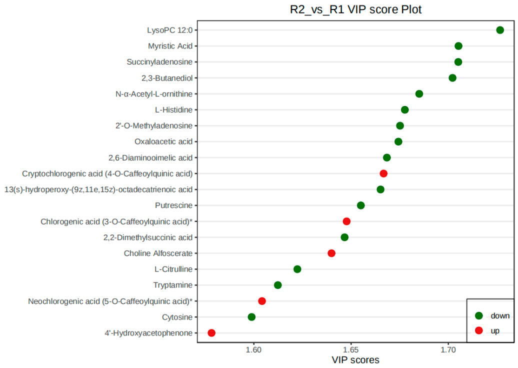

(a) (CK-R vs. LN-R)

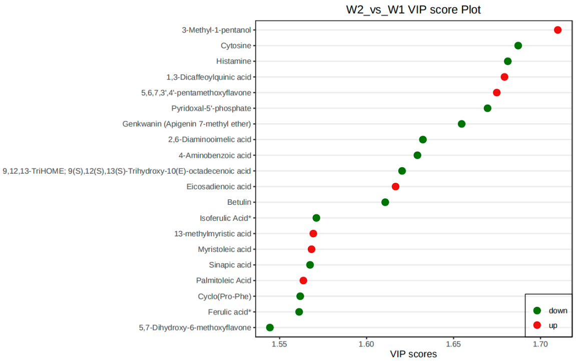

(b) (CK-W vs. LN-W)

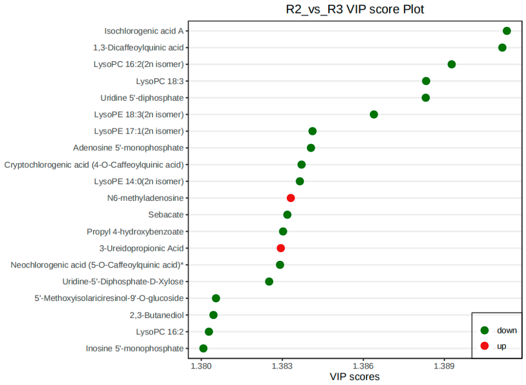

(c) (CK-R vs. HN-R)

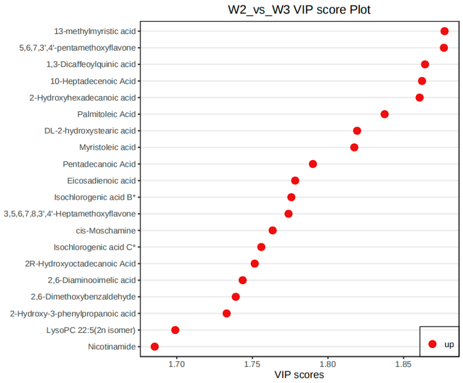

(d) (CK-W vs. HN-W)

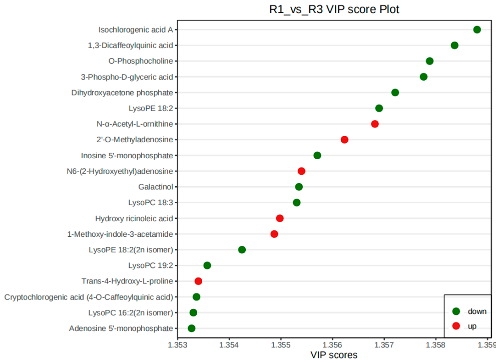

(e) (LN-R vs. HN-R)

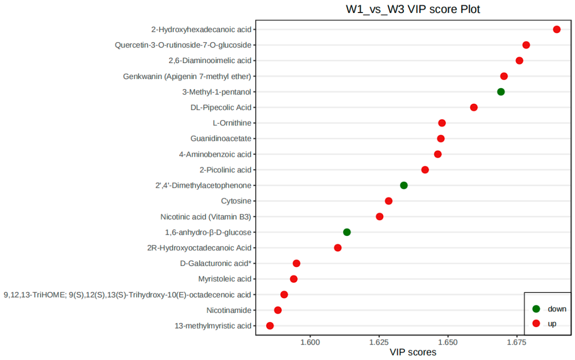

(f) (LN-W vs. HN-W)

FigureS4 (a-e) The VIP value diagram of DEMs; The abscissa represents the VIP value, the ordinate represents the differential metabolite, the red represents the up regulation differential metabolite, and the green represents the down regulation differential metabolite
