# Supplementary material for: Transcriptome and Metabolome Analyses Reveal Mechanisms Underlying the Response of Quinoa Seedlings to Nitrogen Fertilizers
Source: Int J Mol Sci. 2023 Jul 18;24(14):11580. doi: 10.3390/ijms241411580 (PMC10380953; doi:10.3390/ijms241411580)
Supplement: Supplementary file 1 [file ijms-24-11580-s001.zip › Figure S5.pdf]

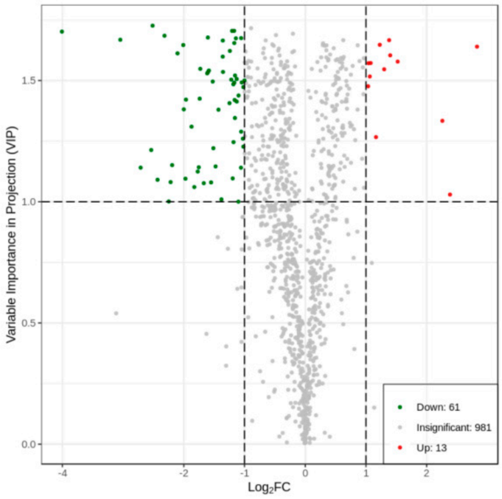

(a) (CK-R vs. LN-R)

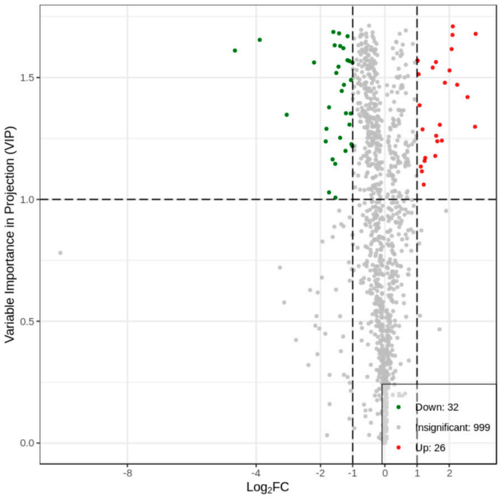

(b) (CK-W vs. LN-W)

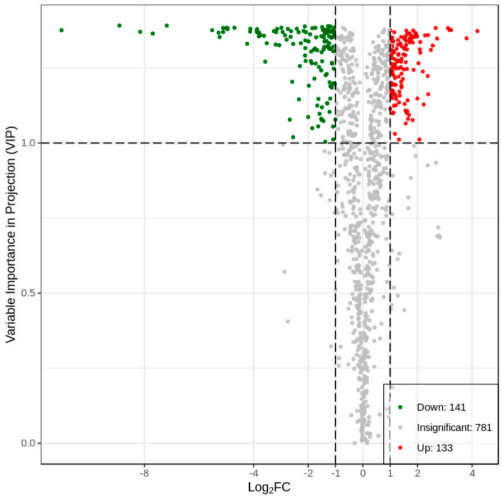

(c) (CK-R vs. HN-R)

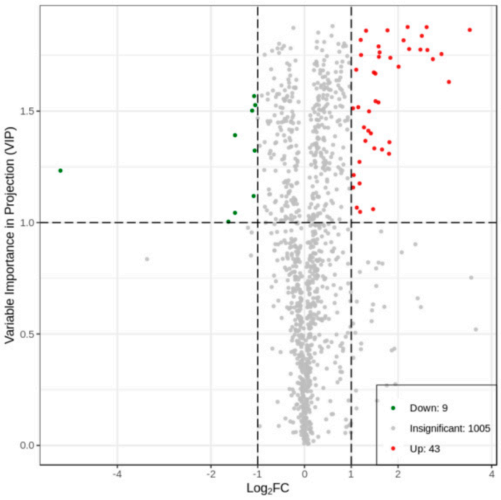

(d) (CK-W vs. HN-W)

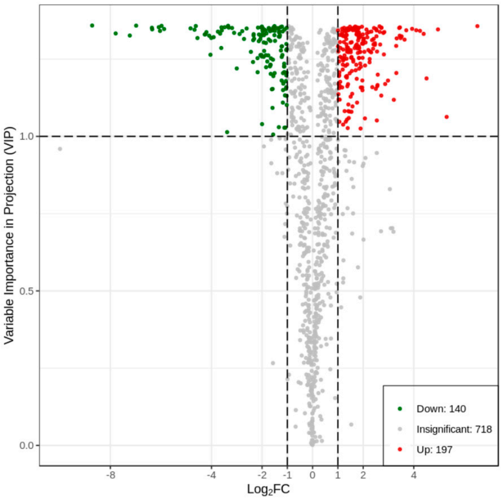

(e) (LN-R vs. HN-R)

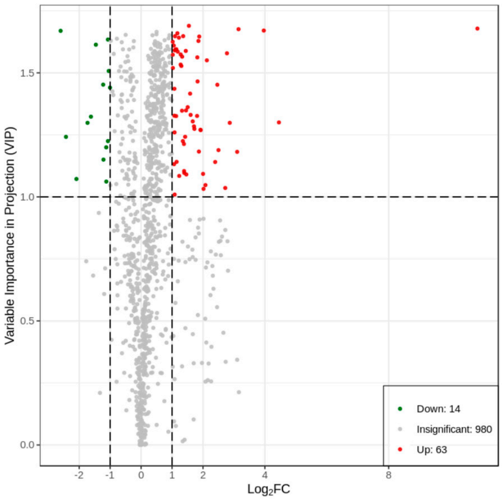

(f) (LN-W vs. HN-W)

Figure S5 Volcanic map of DEMs; (a),(c) and (e) represent LNvs.CK, HNvs.CK and LN vs. HN for red quinoa, respectively; (b), (d) and (e) represent LNvs.CK, HNvs.CK and LN vs. HN for white quinoa, respectively; Each point in the volcano map represents a metabolite, in which the

green point represents the down regulated differential metabolite, the red point represents the up regulated differential metabolite, and the gray point represents the detected but not significantly different metabolite; The abscissa represents the logarithm (log2FC) of the relative content difference multiple of a metabolite in the two groups of samples. The greater the absolute value of the abscissa, the greater the relative content difference between the two groups of samples. Under VIP+FC dual screening conditions: the ordinate represents the VIP value. The larger the ordinate value is, the more significant the difference is, and the more reliable the differential expression metabolites obtained through screening.
