# Supplementary material for: Transcriptome and Metabolome Analyses Reveal Mechanisms Underlying the Response of Quinoa Seedlings to Nitrogen Fertilizers
Source: Int J Mol Sci. 2023 Jul 18;24(14):11580. doi: 10.3390/ijms241411580 (PMC10380953; doi:10.3390/ijms241411580)
Supplement: Supplementary file 1 [file ijms-24-11580-s001.zip › Figure S6.pdf]

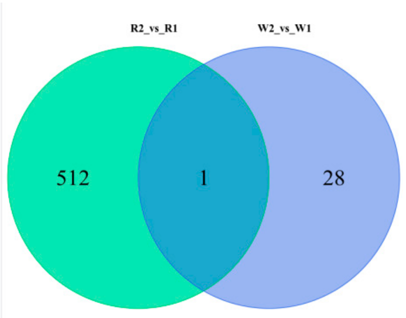

a (CKvs.LN)

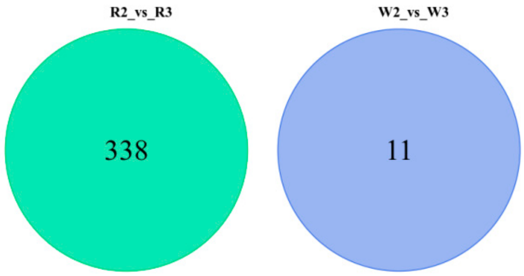

b (CKvs.HN)

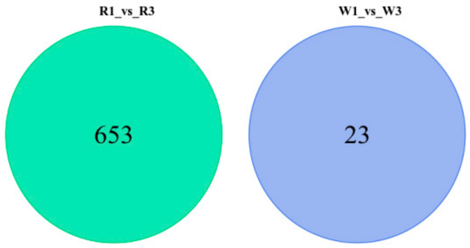

c (LN vs. HN)

Figure S6 (a) Venn diagram of DEGs for CK-Rvs.LN-R and CK-Wvs.LN-W. (b) Venn diagram of DEGs for CK-Rvs.HN-R and CK-Wvs.HN-W. (c) Venn diagram of DEGs for LN-Rvs.HN-R and LN-Wvs.HN-W.
