# Supplementary material for: Transcriptome and Metabolome Analyses Reveal Mechanisms Underlying the Response of Quinoa Seedlings to Nitrogen Fertilizers
Source: Int J Mol Sci. 2023 Jul 18;24(14):11580. doi: 10.3390/ijms241411580 (PMC10380953; doi:10.3390/ijms241411580)
Supplement: Supplementary file 1 [file ijms-24-11580-s001.zip › Figure S7.pdf]

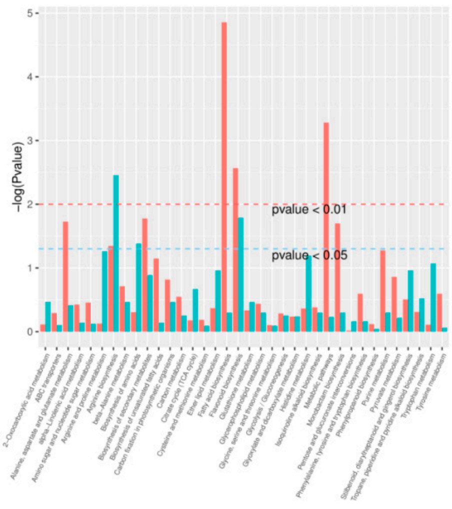


**a (CK-Rvs.LN-R)**

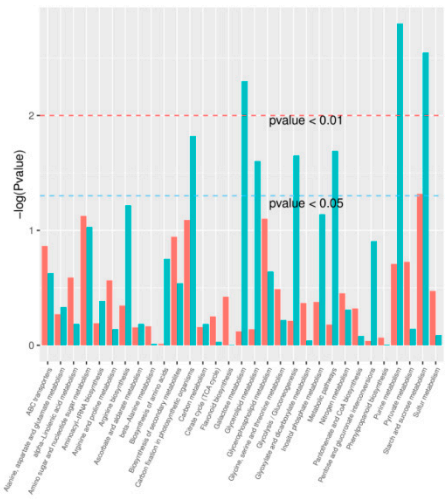


**b (CK-Rvs.HN-R)**

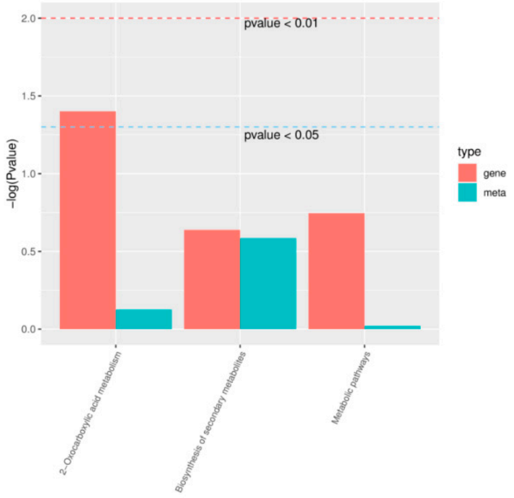


**c (CK-Wvs.LN-W)**

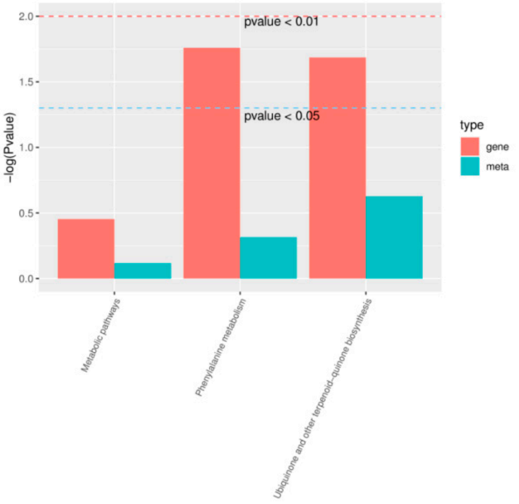


**d (CK-Wvs.HN-W)**

**Figure S7 P-Value histogram of KEGG enrichment analysis;** Demonstrate the enrichment of pathways with both differential metabolites and differential genes. The abscissa of the histogram represents the metabolic pathway, the red represents the enriched pvalue value of the differential gene in the ordinate, and the green represents the enriched pvalue value of the differential metabolite, expressed in log (P-value). The higher the ordinate, the stronger the enrichment degree.
