# Supplementary material for: Transcriptome and Metabolome Analyses Reveal Mechanisms Underlying the Response of Quinoa Seedlings to Nitrogen Fertilizers
Source: Int J Mol Sci. 2023 Jul 18;24(14):11580. doi: 10.3390/ijms241411580 (PMC10380953; doi:10.3390/ijms241411580)
Supplement: Supplementary file 1 [file ijms-24-11580-s001.zip › Table S2.pdf]

Table S2 Commom differential metabolites between red quinoa and white quinoa in LN vs CK

number	Index	Formula	Compounds	Class I	VIP	p_value	Fold_Change	Log2FC	Type
1	pme1816	C16H18O9	Neochlorogenic acid (5-O-Caffeoylquinic acid)*	Phenolic acids	1.60E+00	9.33E-02	2.64E+00	1.40E+00	up
2	pmf0256	C6H14O	3-Methyl-1-pentanol	Others	1.55E+00	8.63E-03	2.46E+00	1.30E+00	up
3	mws0178	C16H18O9	Chlorogenic acid (3-O-Caffeoylquinic acid)*	Phenolic acids	1.65E+00	4.06E-02	2.34E+00	1.23E+00	up
4	pmp000117	C22H24O9	3,5,6,7,8,3',4'-Heptamethoxyflavone	Flavonoids	1.49E+00	1.43E-02	4.45E-01	-1.17E+00	down
5	mws0255	C4H5N3O	Cytosine	Nucleotides and derivatives	1.60E+00	9.52E-03	3.90E-01	-1.36E+00	down
6	pme2122	C5H9N3	Histamine	Alkaloids	1.54E+00	1.26E-02	3.33E-01	-1.59E+00	down
7	mws1336	C7H7NO2	4-Aminobenzoic acid	Phenolic acids	1.08E+00	2.80E-02	3.14E-01	-1.67E+00	down
8	Hmbn005207	C30H48O4	Hederagenin	Terpenoids	1.12E+00	3.27E-01	2.93E-01	-1.77E+00	down
9	Zmyn000155	C7H14N2O3	N-α-Acetyl-L-ornithine	Amino acids and derivatives	1.69E+00	5.23E-02	2.00E-01	-2.32E+00	down
10	pmp000438	C30H50O2	Betulin	Terpenoids	1.21E+00	3.00E-01	1.72E-01	-2.54E+00	down
11	pme0278	C7H14N2O4	2,6-Diaminooimelic acid	Organic acids	1.67E+00	1.30E-02	1.21E-01	-3.05E+00	down
