# Supplementary material for: Transcriptome and Metabolome Analyses Reveal Mechanisms Underlying the Response of Quinoa Seedlings to Nitrogen Fertilizers
Source: Int J Mol Sci. 2023 Jul 18;24(14):11580. doi: 10.3390/ijms241411580 (PMC10380953; doi:10.3390/ijms241411580)
Supplement: Supplementary file 1 [file ijms-24-11580-s001.zip › Table S3.pdf]

Table S3 Common differential metabolites between red quinoa and white quinoa in HN vs CK

Number	Index	Formula	Compounds	Class I	VIP	p_value	Fold_Change	Log2FC	Type
1	mws0885	C7H6O4	2,4-Dihydroxybenzoic acid	Phenolic acids	1.50E+00	2.43E-02	4.60E-01	-1.12E+00	down
2	Zmyn000155	C7H14N2O3	N-α-Acetyl-L-ornithine	Amino acids and derivatives	1.41E+00	2.25E-01	2.57E+00	1.36E+00	up
3	pme0008	C6H13N3O3	L-Citrulline	Amino acids and derivatives	1.16E+00	1.92E-01	2.06E+00	1.04E+00	up
4	mws0361	C16H30O2	Palmitoleic Acid	Lipids	1.84E+00	1.66E-04	5.69E+00	2.51E+00	up
5	pmp001245	C15H22NO4+	Feruloylcholine	Alkaloids	1.54E+00	1.99E-01	2.99E+00	1.58E+00	up
6	MWS2673	C20H34O4	5,6-DiHETrE [5,6-dihydroxy-8Z,11Z,14Z-eicosatrienoic acid]	Lipids	1.51E+00	5.38E-02	2.05E+00	1.04E+00	up
7	MWStz224	C20H20N2O4	N-Feruloylserotonin	Alkaloids	1.33E+00	1.65E-01	3.15E+00	1.66E+00	up
8	MWStz202	C20H20N2O4	cis-Moschamine	Alkaloids	1.76E+00	4.06E-03	3.04E+00	1.60E+00	up
9	pme1816	C16H18O9	Neochlorogenic acid (5-O-Caffeoylquinic acid)*	Phenolic acids	1.21E+00	3.24E-01	2.07E+00	1.05E+00	up
10	mws0178	C16H18O9	Chlorogenic acid (3-O-Caffeoylquinic acid)*	Phenolic acids	1.27E+00	2.77E-01	2.26E+00	1.17E+00	up
11	mws1313	C20H20O7	5,6,7,3',4'-pentamethoxyflavone	Flavonoids	1.88E+00	1.57E-05	6.11E+00	2.61E+00	up
12	HJN086	C21H22O11	Eriodictyol-3'-O-glucoside	Flavonoids	1.32E+00	8.28E-02	4.78E-01	-1.06E+00	down
13	pme2125	C18H32O16	Raffinose	Others	1.53E+00	4.16E-02	4.82E-01	-1.05E+00	down
14	pmn001382	C25H24O12	Isochlorogenic acid A	Phenolic acids	1.63E+00	3.53E-03	8.49E+00	3.09E+00	up
15	MWSmce346	C25H24O12	1,3-Dicaffeoylquinic acid	Phenolic acids	1.86E+00	3.33E-03	1.16E+01	3.53E+00	up
16	Li512115	C25H24O12	Isochlorogenic acid B*	Phenolic acids	1.78E+00	3.35E-03	5.57E+00	2.48E+00	up
17	pmn001384	C25H24O12	Isochlorogenic acid C*	Phenolic acids	1.76E+00	7.06E-03	7.61E+00	2.93E+00	up
18	Lmbn001181	C22H28O15	Fraxetin-7,8-di-O-glucoside	Lignans and Coumarins	1.43E+00	3.38E-02	2.41E+00	1.27E+00	up
19	MWSHY0056	C27H32O14	Naringenin-7-O-Rutinoside(Narirutin)	Flavonoids	1.40E+00	2.09E-01	2.67E+00	1.42E+00	up
20	Hmcp001578	C28H32O17	Isorhamnetin-3,7-O-diglucoside	Flavonoids	1.50E+00	1.31E-01	2.60E+00	1.38E+00	up
21	pmp000117	C22H24O9	3,5,6,7,8,3',4'-Heptamethoxyflavone	Flavonoids	1.49E+00	1.43E-02	4.45E-01	-1.17E+00	down
22	pmb0639	C33H40O20	Apigenin-8-C-glucoside-7-O-Sophoroside	Flavonoids	1.07E+00	3.35E-01	2.17E+00	1.12E+00	up
