# Supplementary material for: Transcriptome and Metabolome Analyses Reveal Mechanisms Underlying the Response of Quinoa Seedlings to Nitrogen Fertilizers
Source: Int J Mol Sci. 2023 Jul 18;24(14):11580. doi: 10.3390/ijms241411580 (PMC10380953; doi:10.3390/ijms241411580)
Supplement: Supplementary file 1 [file ijms-24-11580-s001.zip › Table S4.pdf]

Table S4 Commom differential metabolites between red quinoa and white quinoa in LN vs HN

number	Index	Formula	Compounds	Class I	VIP	p_value	Fold_Change	Log2FC	Type
1	pmf0256	C6H14O	3-Methyl-1-pentanol	Others	1.26E+00	8.20E-03	4.28E-01	-1.22E+00	down
2	mws0255	C4H5N3O	Cytosine	Nucleotides and derivatives	1.19E+00	4.39E-02	2.55E+00	1.35E+00	up
3	pme2122	C5H9N3	Histamine	Alkaloids	1.23E+00	4.29E-02	4.21E+00	2.08E+00	up
4	MWS0811	C6H11NO2	DL-Pipecolic Acid	Organic acids	1.06E+00	2.75E-01	4.19E+00	2.07E+00	up
5	pme2527	C5H12N2O2	L-Ornithine	Amino acids and derivatives	1.34E+00	4.08E-04	1.30E+01	3.70E+00	up
6	pme0040	C5H5N5	Adenine	Nucleotides and derivatives	1.30E+00	1.14E-03	3.36E+00	1.75E+00	up
7	pme0033	C5H4N4O	Hypoxanthine	Nucleotides and derivatives	1.31E+00	1.99E-02	5.10E+00	2.35E+00	up
8	mws0254	C6H9N3O2	L-Histidine	Amino acids and derivatives	1.32E+00	1.92E-02	3.61E+00	1.85E+00	up
9	MWS0559	C6H10O5	1,6-anhydro-β-D-glucose	Others	1.17E+00	1.04E-01	4.47E-01	-1.16E+00	down
10	Zmyn000155	C7H14N2O3	N-α-Acetyl-L-ornithine	Amino acids and derivatives	1.36E+00	1.70E-02	9.15E+01	6.52E+00	up
11	mws0260	C6H14N4O2	L-Arginine	Amino acids and derivatives	1.33E+00	1.68E-02	2.10E+01	4.39E+00	up
12	pme0278	C7H14N2O4	2,6-Diaminooimelic acid	Organic acids	1.31E+00	6.12E-02	1.24E+01	3.64E+00	up
13	pme2743	C10H11NO3	N-Phenylacetylglycine	Amino acids and derivatives	1.30E+00	6.99E-02	5.52E+00	2.46E+00	up
14	mws1189	C6H10O7	D-Galacturonic acid*	Organic acids	1.23E+00	7.22E-03	3.31E+00	1.73E+00	up
15	Lmlp003161	C14H20N2O3	N-Feruloylputrescine	Alkaloids	1.05E+00	2.42E-01	5.81E+00	2.54E+00	up
16	pmp001245	C15H22NO4+	Feruloylcholine	Alkaloids	1.30E+00	9.70E-02	6.37E+00	2.67E+00	up
17	ML10195036	C18H37NO2	3-Dehydrosphinganine	Lipids	1.33E+00	5.82E-04	3.41E+00	1.77E+00	up
18	Lmbn003970	C18H34O5	9,12,13-TriHOME; 9(S),12(S),13(S)-Trihydroxy-10(E)-octadecenoic acid	Lipids	1.35E+00	6.03E-03	2.67E+00	1.42E+00	up
19	MWStz202	C20H20N2O4	cis-Moschamine	Alkaloids	1.17E+00	8.50E-02	5.93E+00	2.57E+00	up
20	Lmdp003509	C20H18O11	Quercetin-3-O-xyloside (Reynoutrin)	Flavonoids	1.24E+00	3.43E-02	3.69E+00	1.88E+00	up
21	pmp000438	C30H50O2	Betulin	Terpenoids	1.06E+00	1.77E-01	3.94E+01	5.30E+00	up
22	pme2125	C18H32O16	Raffinose	Others	1.15E+00	6.52E-02	3.27E-01	-1.61E+00	down
23	mws1409	C15H22N2O18P2	Uridine-5'-diphosphoglucuronic acid	Nucleotides and derivatives	1.26E+00	1.44E-02	2.45E-01	-2.03E+00	down
24	Hmlp002975	C28H30O17	Isorhamnetin-3-O-glucuronide-7-O-rhamnoside	Flavonoids	1.30E+00	4.68E-02	4.26E+00	2.09E+00	up
25	Hmcp001578	C28H32O17	Isorhamnetin-3,7-O-diglucoside	Flavonoids	1.25E+00	5.04E-02	2.31E+00	1.21E+00	up
26	Lmsp003252	C28H32O17	Isorhamnetin-3-O-sophoroside	Flavonoids	1.28E+00	5.89E-02	2.84E+00	1.51E+00	up
27	Lmmp002334	C33H40O21	Quercetin-3-O-rutinoside-7-O-glucoside	Flavonoids	1.33E+00	9.59E-03	3.55E+00	1.83E+00	up
28	Hmmp006067	C42H68O13	Oleanolic acid-3-O-glucosyl(1→2)glucoside	Terpenoids	1.12E+00	1.05E-01	9.26E+00	3.21E+00	up
29	pmb0620	C34H42O21	Chrysoeriol-6,8-di-C-glucoside-7-O-glucoside	Flavonoids	1.20E+00	1.21E-02	2.56E+00	1.36E+00	up
