# Supplementary material for: Transcriptome and Metabolome Analyses Reveal Mechanisms Underlying the Response of Quinoa Seedlings to Nitrogen Fertilizers
Source: Int J Mol Sci. 2023 Jul 18;24(14):11580. doi: 10.3390/ijms241411580 (PMC10380953; doi:10.3390/ijms241411580)
Supplement: Supplementary file 1 [file ijms-24-11580-s001.zip › Table S5.pdf]

Table S5 Data quality statistics of quinoa transcriptome

Sample	Total Reads	Reads mapped	Unique mapped	Multi mapped	Q30(%)	GC Content(%)
R1-4	50196648	47694939(95.02%)	42046202(83.76%)	15242801(11.25%)	93.9	43.31
R1-5	51332586	49422483(96.28%)	40858695(79.60%)	26669283(16.68%)	94.51	43.45
R1-6	46839506	43977583(93.89%)	35881301(76.60%)	23388211(17.29%)	92.81	43.29
R2-4	48569484	46944266(96.65%)	42492053(87.49%)	11400537(9.17%)	94.58	43.39
R2-5	46363050	44390962(95.75%)	40139891(86.58%)	11068438(9.17%)	91.81	43.57
R2-6	54550166	47319494(86.74%)	35086377(64.32%)	32912906(22.43%)	94.48	45.58
R3-4	41087168	38541570(93.80%)	26897339(65.46%)	36849033(28.34%)	91.89	43.78
R3-5	44650396	43102840(96.53%)	39550539(88.58%)	8404828(7.96%)	94.32	44.29
R3-6	43852582	42181445(96.19%)	37686481(85.94%)	11998437(10.25%)	94.12	43.96
W1-4	49174948	46847221(95.27%)	43483023(88.43%)	7635347(6.84%)	93.47	43.49
W1-5	49075774	46835697(95.44%)	40421003(82.36%)	17026942(13.07%)	94.64	43.56
W1-6	53796560	50917334(94.65%)	42754689(79.47%)	22571047(15.17%)	94.26	43.46
W2-4	44246504	35500134(80.23%)	32655171(73.80%)	5836412(6.43%)	92.33	45.87
W2-5	51326632	49154482(95.77%)	45500170(88.65%)	8605022(7.12%)	94.13	43.41
W2-6	53489678	51026476(95.39%)	45448734(84.97%)	12799980(10.43%)	94.19	43.61
W3-4	52173672	49357885(94.60%)	43282963(82.96%)	15283985(11.64%)	93.94	43.35
W3-5	66907910	62572767(93.52%)	57776333(86.35%)	10854957(7.17%)	94.12	42.77
W3-6	42814612	42006998(98.11%)	38685792(90.36%)	5184662(7.76%)	92.51	47.20
